# Role of Dietary and Environmental Factors on Thyroid Cancer in Romania: A Brief Review

**DOI:** 10.3390/diagnostics12081959

**Published:** 2022-08-13

**Authors:** Gilles Kermoison, Ciprian Draganescu

**Affiliations:** 1 Doctoral School of Iuliu Hațieganu, University of Medicine and Pharmacy, 400012 Cluj-Napoca, Romania; 2 Nuclear Medicine Department, “Prof. Dr. Ion Chiricuta” Institute of Oncology, 400015 Cluj-Napoca, Romania; 3 Nuclear Medicine Department, University Institut of Martinique, 97200 Fort-de-France, France

**Keywords:** thyroid neoplasm, environmental factors, dietary, incidence, Romania

## Abstract

Thyroid cancer is the most common endocrine tumor, accounting for about 1% of all human malignancies. There are environmental factors that can potentiate the onset of thyroid cancer, in particular pollutants, lifestyle or radiation exposure. Another major cause responsible for the appearance of thyroid cancer is the habitat in endemic areas where there is a deficit of iodine in the soil, drinking water and food. We operated using the PubMed database in order to find the articles of interest. After a wary review of the literature, we designated the relevant articles necessary for our study including various factors such as alimentation, effects of the Chernobyl fallout radiation and the iodine and vitamin D deficiency in Romania. The aim of this article is to make a correlation between the different environmental and dietary factors in Romania, and the increased incidence of thyroid cancer.

## 1. Introduction

Among cancers, thyroid carcinoma (TC) is the most common endocrine tumor with the largest annual incidence, accounting for 3.4% of all cancers diagnosed annually [1]. The incidence of thyroid carcinoma is increasing due to better diagnostic techniques but also to exposure to genetic and environmental factors [2,3]. In the Oncological Institute “Prof. Dr. Ion Chiricuță”, Cluj-Napoca reported an increase in the incidence of thyroid cancer after the Chernobyl explosion from two new cases per year in 1970 to 553 new annual cases in 2012, respectively, and 745 new cases in 2015 [3]. Thyroid cancer is the site with the widest rise, from 3.71 to 11.7 cases /100,000 in Cluj during the period of 2000 to 2013 [4]. There are four types of thyroid cancer: papillary thyroid carcinoma and follicular thyroid carcinoma, representing differentiated thyroid carcinoma, undifferentiated thyroid carcinoma and medullary thyroid carcinoma [5]. Papillary, follicular and undifferentiated thyroid carcinoma hail from the thyroid follicular epithelial cells, while medullary thyroid carcinoma derives from the parafollicular cells. Differentiated thyroid cancers represent approximately 90% of all thyroid cancers [6]. Undifferentiated thyroid carcinoma is a rare tumor associated with aggressive behavior and short median time of survival [1]. 

Several studies report that environmental factors and lifestyle could directly contribute to this carcinoma. Thus, we performed a comprehensive review of our environmental factors of interest significantly associated with thyroid carcinogenesis. A better mastery of environmental and dietary knowledge is a very important tool for cancer prevention through true risks prevention and management.

## 2. Materials and Methods

The international database PubMed was used to identify articles and studies that have evaluated the correlation between environmental and dietary factors and thyroid cancer.

We defined our search strategy by applying the following combinations of keywords: thyroid cancer with iodine deficiency, vitamin D deficiency, dietary pattern and Chernobyl, ultimately obtaining 1590 articles. We adjusted the search filters adding “full text” and “English”, retaining a selection of 1084 articles. An additional filter was applied to include articles on the Romanian population. Furthermore, we analyzed the bibliography of each article and added five more studies, four of which were found on the Google Scholar database and another through ResearchGate. The following exclusion criteria were applied: studies published before 2000, abstract without available full text and reviews.

**Figure 1:**
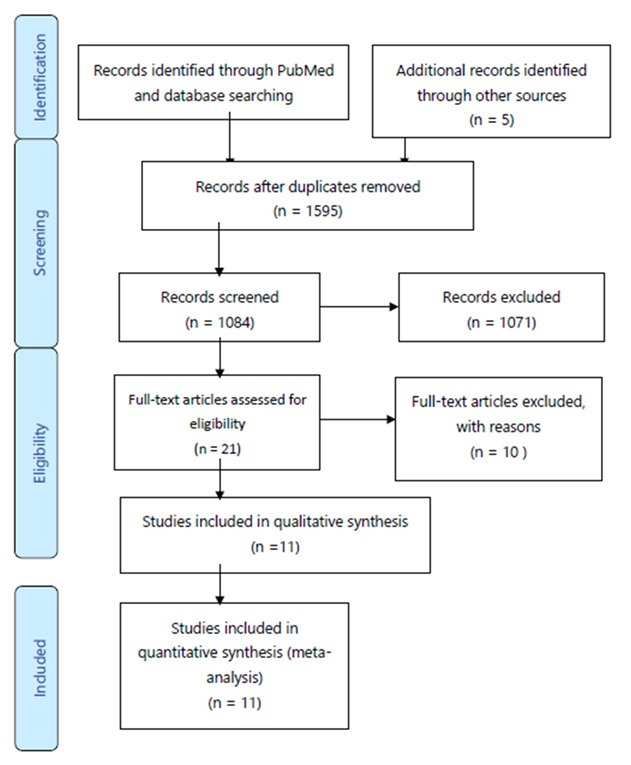
PRISMA flow diagram of selected studies.

Finally, we kept a selection of 10 articles published between 2000 and 2022. The PRISMA flow diagram is presented in Figure 1.

## 3. Results

During our search of the available literature while applying our established criteria, we found four articles showing a link between thyroid cancer and iodine deficiency in Romania (Table 1 and Table 2). 

A very reduced number of studies were found concerning dietary pattern and thyroid cancer, and, after the additional filter, none were identified concerning Romania. A considerable number of articles showing the association between vitamin D deficiency and the potential risk factor for thyroid cancer were found and two articles about the vitamin D status in Romania were selected. The search of articles showing the association between vitamin D deficiency and thyroid cancer in Romania was inconclusive. We found 10 articles regarding the effects of the Chernobyl fallout on the Romanian population, of which two were common with those of Table 1 (Table 3 and Table 4). We introduced as well a summary of quality assessment of the included studies: QUADAS-2 (Table 5).

## 4. Discussion

### 4.1. Iodine Deficiency

Recommended daily iodine intakes from WHO/UNICEF/ICCIDD are 90 μg for infants and young children <5 years, 120 μg for children 6–12 years, 150 μg for adolescents and adults, and 250 μg for pregnant and lactating women [16]. 

Considering that more than 90% of dietary iodine appears in the urine, the urinary iodine concentration is a biomarker of recent iodine intake and is the recommended indicator for the evaluation of iodine status in populations (Table 6) [17]. 

Iodine is necessary for human life and represents a crucial trace element for thyroid function. Multiple reports confirm that populations with iodine deficiency developed goiter and nodularity, which, in many cases, preceded the development of thyroid cancer. Moreover, in that specific population, there is a prevalence for thyroid follicular cancers.

Iodine deficiency is a major problem in Romania and almost the entire population of the country is at risk. Beyond 35% of the population shows visible signs of goiter and nearly 20% of children are iodine deficient. The higher prevalence of iodine deficiency disorders was found in rural areas [8,18].

The human organism requires continuous, very low amounts of iodine of around 200 micrograms per day; this can be reached by adding iodine to salt. Salt is universally consumed and in equal quantities by the entire population, and it represents a good vector for iodine intake [19]. 

In 2002, nationwide surveys demonstrated that non-iodized salt is still present in Romania: 31% in urban areas and 37% in rural areas. The Romanian population that exclusively uses iodized salt is represented based on the following: 53% in rural areas and 56% of the households in urban areas. The highest percentage of the population using exclusively iodized salt is found in Bucharest, with a rate of 71% [19]. 

C. Buzduga et al. [7] correlated the incidence and the histology of thyroid cancer in Moldova (Romania), which is a mild endemic goiter zone, between 2001 and 2004, with the incidence and the histology of thyroid cancer between 2005 and 2008 in the same region after the introduction of iodized salt.

The number of thyroid cancers has raised after the introduction of iodized salt, with 178% compared to 2001–2004, even if the number of thyroidectomies decreased from 1734 (2001–2004) to 1449 (2005–2008) [7]. 

Some studies attribute the rise in thyroid cancer to the frequent use of ultrasonography and fine needle biopsy [20], but this is unlikely to account for the observed increase in the total number of thyroid cancers from this study because the diagnostic methods remained the same as the ones reported by the study made in 2001–2004 in the Endocrinology Clinic from Saint Spiridon Hospital Iasi. 

Z. Szántó et al. [8] showed that the papillary/follicular carcinomas ratio presented a gradual increase between 1984 and 2007, despite the fact that a descending trend in follicular thyroid cancer frequency has not yet attained significant values. This can be explained by the latency time of well-differentiated thyroid carcinomas, which can take several years. The effect of universal iodization on thyroid cancer in Mures County has not been fully elucidated, but it seems that there has been a decrease in the frequency of follicular carcinomas and an increase in the papillary type of TC [8]. 

Iodine prophylaxis in an iodine-deficient population shows a tendency to decrease follicular thyroid carcinoma but lead to a predominant papillary histotype [9,21,22]. 

This supports the conjecture that iodine deficiency is associated with an increased risk of follicular thyroid carcinoma, while high iodine intake may increase the risk of papillary thyroid carcinoma [6,22], more aggressive histological tumor characteristics [23], and increased risk for thyroid nodules [10].

Furthermore, Kim et al. [24] concluded that BRAF mutations in papillary thyroid carcinoma were recurring in subjects with low or excessive iodine intakes. 

Iodine deficiency may lead to the hypersecretion of the thyroid-stimulating hormone (TSH) by decreasing thyroid hormone production, which, in terms of the hypertrophy and hyperplasia of the thyroid follicular cells, may potentiate the onset of cancer [25].

### 4.2. Dietary Pattern

Many epidemiological studies indicate an increased incidence of thyroid carcinoma, mostly concerning the papillary histotype, suggesting that some carcinogens might stimulate molecular modifications. Eating habits represent one of the most discussed elements.

Fish consumption is an essential source of iodine and other micronutrients; however, it can contain several pollutants. The prospective study European Prospective Investigation into Cancer and Nutrition (EPIC) assured that the consumption of fish is not connected with an increased risk of thyroid carcinoma [26].

Romanians consume less fish than other countries in the European Union and are more exposed to diseases, especially diseases related to poor diet, than other EU citizens. Approximately, Romanians consume 7 kg of fish yearly, while EU citizens consume 24 kg [27]. 

The link between fruits and vegetables consumption and thyroid carcinoma was explored by Zamora-Ros et al. using data from the EPIC cohort [28]. Their study results corroborated that there is no association between thyroid carcinoma and fruits or vegetables consumption. In 2014, 65.7% of the European population over the age of 15 were reported to eat at least one portion of fruits and vegetables every day. In Romania, it represented less than 50% of the population [29]. 

Goitrogens are substances that disrupt the production of thyroid hormones by interfering with iodine uptake in the thyroid gland [18]. The main goitrogen foods are vegetables in the cruciferous category. Some of the more common and potent goitrogens include the following types of food: African cassava, broccoli, brussels sprouts, cabbage, cauliflower, kale, mustard, peaches, peanuts, radishes, spinach, strawberries and watercress [18].

A recent epidemiological review summarizing the various studies on nutritional factors and thyroid cancer concluded that a high consumption of cruciferous vegetables was inversely associated with thyroid cancer risk, but the results were very heterogenic depending on the countries [30].

The consumption of soy-based foods and alfalfa sprouts was related to thyroid cancer risk reduction [21].

In the EPIC study, the association between alcohol consumption and differentiated thyroid cancer was also inspected [31]. In comparison to other studies reporting an elevated risk of cancer with alcohol consumption, this study indicated that the consumption of 15 or more grams daily had a 24% lower risk of differentiated thyroid carcinomas compared to individuals consuming 0.1–4.9 g of alcohol. Regardless, the mechanisms explaining the association between alcohol consumption and thyroid cancer risk are still unclear. Compared to EU new members, Romania has a good rate of abstainers for men and women (second place after Bulgaria). On the other hand, the prevalence of heavy drinkers is worrying, Romanian women are the 3rd highest alcohol consumers in the EU, and Romanian men are in 5th position [32]. 

Recent diverse studies have proven a relationship between consuming macronutrients and tumor susceptibility. Those mechanisms have not yet been totally defined; however, it has been suggested that carbohydrates enhance insulin resistance and increase the risk of developing cancer. The link between macronutrients, namely carbohydrate and protein consumption, and differentiated thyroid cancer risk have lately been studied [30]. It showed that for women, a higher risk of thyroid cancer is associated with excessive caloric consumption of protein and carbohydrates [33]. A carbohydrate-rich diet represents a risk factor for the expansion of insulin resistance, which has been associated with thyroid cancer development [34]. 

Nitrite and nitrate absorption by water or food has been considered to increase the risk of differentiated thyroid cancer in several studies [35,36]. In fact, nitrate can be affiliated to nitrate’s specific inhibition of iodide uptake by the thyroid. A reduction in intra-thyroidal iodide can produce a lower fabrication of thyroid hormones and increase TSH levels. TSH stimulation of the thyroid represents a major risk factor in the onset of thyroid carcinoma [37]. 

Before 1989, the Romanian government asked for an important land fertilization process in order to expand agricultural production, disregarding environmental risks and the repercussion on human health. Throughout this period, the authorities noticed an intensification of nitrates in soil and water. 

After 1989, national and international agencies became more concerned about the general pollution of the Romanian environment. According to estimates, 1.5 million people were possibly exposed to nitrates from rural wells [38].

### 4.3. Vitamin D Deficiency 

Sun exposure is the main source of vitamin D, even in countries where biofortification is adopted [4]. Adequate dietary and supplemental vitamin D consumption, together with reasonable sun exposure (around 5–10 min of exposure two or three times weekly), are efficient methods for assuring vitamin D sufficiency [16]. In Romania, vitamin D supplementation is practiced mostly for children under 1 year. 

Many studies have estimated the impact of vitamin D in the pathogenesis of thyroid dysfunctions [39], although the relationship between vitamin D deficiency and thyroid cancer remains questionable. Some studies have approved the fact that high vitamin D levels could protect against thyroid cancer, while others revealed that reduced vitamin D levels are related to an increased risk of thyroid cancer and aggressiveness [40].

Sahin et al. [41] noted that 344 patients with papillary thyroid cancer had significantly lower 25(OH)D levels than 116 controls, and vitamin D insufficiency (25(OH)D level <50 nmol/L) was more prevalent in this group.

M Roskies et al. [42] studied the 25-hydroxyvitamin D (3) levels of 212 patients before their thyroidectomy and the patients were ranked based on vitamin D status. Indeed, vitamin D deficiency showed levels under the established line of 37.5 nmol/L and vitamin D sufficiency reflected levels over it. The malignancy growth rate, when comparing the vitamin D sufficiency and vitamin D deficiency groups, from 37.5% to 75%, respectively, corresponds to a relative risk of 2.0, explaining that vitamin D deficiency may be a risk factor for thyroid cancer.

Kim et al. [43] reported 548 female patients who had total thyroidectomy for papillary thyroid carcinoma. The preoperative 25(OH)D levels were significantly reduced in patients with a tumor size of >1cm or lymph node metastasis. 

Stepien et al. [44] showed decreased 1,25(OH)2D3 levels in 27 papillary cancer patients (16 follicular cancer patients and 7 anaplastic patients) when compared to 26 healthy controls.

Jonklaas et al. [45] explained that there is no link between preoperative 25(OH)D levels and the diagnosis of thyroid cancer or prognostic and disease stage.

Danilovic et al. [46], investigated 433 patients who had a thyroidectomy and observed no significant distinction in preoperative 25(OH)D levels.

A small number of data are available on the clinical interest of vitamin D supplementation as a procedure to lower the risk of cancer. Few in vitro and animal studies have indicated that important concentrations could reduce the progression of the cell cycle and induce apoptosis.

In 2014, A Chirita-Emandi et al. [47] studied 6631 individuals from across Romania between 2012 and 2014 and showed that vitamin D levels increased from April to September and diminished from October to March. Men, compared to women, showed higher percentages of sufficiency.

Vitamin D levels were ranked as severe deficiency < 10 ng/mL, deficiency 10–20 ng/mL, insufficiency 21–29 ng/mL, sufficiency ≥ 30 ng/mL and potentially harmful > 100 ng/mL. 

Overall, 40% presented sufficient vitamin D, while the rest were insufficient 33%, deficient 22%, severely deficient 4% and 1% potentially harmful [40].

In 2017, D Niculescu et al. [48] measured serum vitamin D in 8024 Romanian subjects and found a marked seasonal variation, with the highest levels in September and the lowest levels in March, similar results comparative to the study of A Chirita-Emandi et al. in 2014.

The prevalence of vitamin D deficiency is high, especially among elderly people. There exists an important seasonal variation in serum 25(OH)D in the whole Romanian population, with the highest levels in September and the lowest levels in March.

### 4.4. Effects of the Chernobyl Fallout Radiation

With the Chernobyl nuclear fallout to blame, thyroid cancer has been one of the cancers with the greatest increase in cases and radioactivity. After this disaster, Romania was exposed to the radioactive clouds and the contamination was heterogeneous in the country. Transylvania and mountain regions showed the highest levels due to the radioactive clouds and raindrops, with an accumulation of radionuclides deposits in soil, water and the biosphere [49].

The two major radioisotopes that were released were 131 Iodine, which has a half-life of 8 days, and 137 Cesium, which has a half-life of 30 years. The main source of radiation responsible for the thyroid cancers seems to be the former [5]. 

Piciu et al. [13] showed a major expansion of thyroid carcinoma cases in Romania since 1970, with a peak of 511% between 2001 and 2010 [50]. They also studied 72 children with thyroid carcinoma treated between 1991 and 2010. The incidence of this cancer increased significantly 10 years after Chernobyl; the growth was exponential, until it reached stability in terms of cases 25 years after the disaster. 

Stefan et al. [11] noted that the majority of affected children with TC were born mostly after the Chernobyl disaster, with many cases recorded among children born in 1996, 1999 and 2000, respectively, at 10, 13 and 14 years after the accident.

Diop et al. showed that children born between 10 and 15 years after the disaster are the most affected, representing 65% of cases [12]. 

However, some studies claim that there was no effect on children born after this catastrophe. 

Davidescu et al. [51] reported that there is no radiation correlated with the incidence rise concerning unborn children at the time of the disaster.

Szanto et al. [8] explained that the incidence of thyroid cancer remained practically the same during 1984–1991 and started to increase continuously from 1992. Two periods of increase have been highlighted in Mures County, the first between 1992 and 1999, caused by the emergence of newly diagnosed papillary thyroid carcinomas, and another between 2000 and 2007, which may be determined by the development of diagnostic methods, such as thyroid ultrasonography.

Catana et al. [9] found an increase in incidence between 1990 and 2009 of 2–5 times, essentially due to papillary thyroid neoplasm.

Likewise, other authors reported a rise in thyroid cancer cases probably caused by high-resolution thyroid ultrasonography and the frequent use of fine-needle aspiration biopsy [52]. Thus, microcarcinomas are detected more frequently in imaging studies. 

During the last three decades, the tumor stages have not diminished, which means that the incidental detection of thyroid carcinomas could not be the only cause of the rising incidence of thyroid cancer [53]. 

Analyzing the incidence of thyroid cancer in Sibiu between the years 2011 and 2013, Stanciu et al. [15] found an increase from 3.98 to 100,000 inhabitants in 2011 to 6.91 to 100,000 in 2013. 

All Romanian studies about thyroid carcinoma incidence showed an increased incidence of 2–5 times depending on each author. The peak of radiogenic thyroid carcinoma appeared within 5–10 years of the disaster, with an important potential of 1–20 years, depending on the exposure rate. Data from Teodoriu et al. [14] complete these results, showing a constant increase in thyroid carcinoma over 30 years after the Chernobyl fallout.

## 5. Conclusions

Thyroid cancer is a problem of global concern, mainly due to the constant rise in incidence from year to year. Epidemiological data are influenced by more accurate methods of diagnosis. Additional studies must be carried out on this matter to clearly see how environmental factors influence this population. In fact, an epidemiological comparative study between Romania and a totally different population in terms of radiation exposure, dietary pattern, climate, iodine and vitamin D deficiency may be useful to study the importance of these factors.

## Figures and Tables

**Table 1:** Studies on iodine deficiency related to thyroid carcinoma in Romania.

Author	Year	Counties
Buzduga et al. [7]	2011	Moldova Romania
Szántó et al. [8]	2009	Mures County
Catana et al. [9]	2012	Mures County
Gaengler et al. [10]	2017	Romania and Cyprus

**Table 2:** Main findings from the case studies on iodine deficiency related to thyroid carcinoma in Romania.

Author	Number of Patients	Main Findings
Buzduga et al. [7]	601	After the introduction of universal iodination: -the number of thyroid cancers has raised. -the number of follicular cancers decreased.
Szántó et al. [8]	288	The universal iodine prophylaxis might increase the papillary/follicular carcinoma ratio.
Catana et al. [9]	524	Increasing proportion of papillary thyroid carcinoma after iodine prophylaxis
Gaengler et al. [10]	208	Participants with inadequate UI (<100 µg/L) had increased risk for thyroid nodules.

**Table 3:** Studies on radiation-induced thyroid carcinoma in Romania due to the Chernobyl nuclear fallout.

Author	Year	Counties
Piciu et al. [3]	2013	Not specified
Szántó et al. [8]	2009	Mures County
Catana et al. [9]	2012	Mures County
Stefan et al. [11]	2020	Not specified
Diop et al. [12]	2019	Not specified
Piciu et al. [13]	2013	Not specified
Teodoriu et al. [14]	2021	Northeast region of Romania
Stanciu et al. [15]	2015	Sibiu

**Table 4:** Main finding from the case studies radiation induced thyroid carcinoma in Romania due to the Chernobyl nuclear fallout.

Author	Number of Patients	Main Findings
Piciu et al. [3]	4779	Rising TC incidence and major increase in the number and aggressiveness of pediatric TC cases
Szántó et al. [8]	288	Incidence of TC started to increase continuously from 1992
Catana et al. [9]	524	Increasing incidence between 1990 and 2009 of 2–5 times, mostly due to papillary thyroid neoplasm
Stefan et al. [11]	62	The majority of affected children with TC were born mostly after Chernobyl disaster, with many cases recorded among children born in 1996, 1999 and 2000 at 10, 13 and 14 years after the accident
Diop et al. [12]	40	Children born between 10 and 15 years after the Chernobyl disaster are the most affected by TC
Piciu et al. [13]	72	Increasing incidence of adult and pediatric TC
Teodoriu et al. [14]	1159	Constant increase in TC over 30 years after the Chernobyl fallout
Stanciu et al. [15]	61	Increasing incidence of TC

**Table 5:** A summary of quality assessment of the included studies: QUADAS-2.

Study	Risk of Bias (QUADAS-2)				Applicability Concerns (QUADAS-2)		
	P	I	R	FT	P	I	R
Piciu et al., 2013 [3]	✓	✓	✓	?	✓	✓	✓
Buzduga et al., 2011 [7]	✓	✓	✓	?	✓	✓	✓
Szántó et al., 2009 [8]	✓	✓	✓	?	✓	✓	✓
Catana et al., 2012 [9]	✓	✗	✓	?	✓	✓	✓
Gaengler et al., 2017 [10]	✗	✓	✓	?	✓	✓	✓
Stefan et al., 2020 [11]	?	✓	✓	?	✓	✓	✓
Diop et al., 2019 [12]	✗	✓	✓	?	✓	✓	✓
Piciu et al., 2013 [13]	✓	✓	✓	?	✓	✓	✓
Teodoriu et al., 2021 [14]	✓	✓	✓	?	✓	✓	✓
Stanciu et al., 2015 [15]	✗	✓	✓	?	✓	✓	✓

P = patient selection; I = index test; R = reference standard; FT = flow and timing. ✓ indicates low risk; ✗ indicates high risk; ? indicates unclear risk.

**Table 6:** Epidemiological criteria for the evaluation of iodine nutrition in a population based on median urinary iodine concentration in school-age children [17].

Median Urinary Iodine (µg/L)	Iodine Intake	Iodine Nutrition
<20	Insufficient	Severe iodine deficiency
20–49	Insufficient	Moderate iodine deficiency
50–99	Insufficient	Mild iodine deficiency
100–199	Adequate	Optimal
200–299	More than adequate	Risk of iodine- induced hyperthyroidism
≥300	Excessive	Risk of adverse health consequences (Iodine-induced hyperthyroidism, autoimmune thyroid disease)
